# A novel blood collection device stabilizes cell-free RNA in blood during sample shipping and storage

**DOI:** 10.1186/1756-0500-6-380

**Published:** 2013-09-26

**Authors:** Jianbing Qin, Thomas L Williams, M Rohan Fernando

**Affiliations:** 1 Research & Development Division, Streck, Inc., 7002 S 109 Street, La Vista, NE 68128, USA; 2 Methodist Hospital Laboratory, 8303 Dodge Street, Omaha, NE 68114, USA

**Keywords:** Clinical laboratory techniques, Blood collection device, Cell-free RNA stability, Blood preservation, Real-time polymerase chain reaction

## Abstract

**Background:**

Cell-free RNA (cfRNA) naturally occurs in blood and has clinical significance. Accurate quantification of these extracellular RNAs in whole blood is hindered by the simultaneous unintended release of cellular RNA and degradation of cfRNA after blood draw. An appropriate blood collection device is needed to stabilize cfRNA during blood processing, transportation and storage, which will ensure cfRNA test reliability. In this study we compared a novel blood collection device against traditional K_3_EDTA tubes for its ability to stabilize cfRNA in blood when subjected to conditions that can occur during sample storage and shipping.

**Findings:**

Shipping blood samples drawn into K_3_EDTA tubes showed a significant increase in mRNA copy numbers for β-actin, c-fos, and 18S rRNA in plasma. In contrast, shipping blood drawn into Cell-Free RNA BCT™s (BCTs) showed only a slight change in mRNA copy numbers for circulating β-actin, c-fos, and 18S rRNA. Moreover, blood stored in K_3_EDTA tubes at 6°C, 22°C and 30°C for 3 days showed a significant increase in mRNA copy numbers for c-fos and β-actin, whereas samples stored in BCTs only showed a slight increase.

**Conclusion:**

Our results show that BCTs minimize increases in background RNA levels caused by temperature fluctuations or agitation that can occur during blood sample storage and shipping. This novel blood collection tube could provide a method for obtaining high quality stabilized cfRNA samples for rare RNA target detection and determining accurate cfRNA concentrations.

## Background

The existence of cell-free nucleic acids in blood has been known for a long time [1]. Evidence accumulated in recent decades indicates that changes in the levels of circulating nucleic acids are associated with certain disease conditions and could be useful for clinical applications, particularly for cancer patients and pregnant women [2]. In 1999, the circulating cell-free RNAs (cfRNAs) were first detected in the plasma or serum of patients with nasopharyngeal carcinoma [3] and malignant melanoma [4]. Later, the investigation was expanded to patients with breast cancer [5], colorectal cancer, follicular lymphoma [6], and hepatocellular carcinoma [7]. Subsequent to the discovery of cfRNA in cancer patients, circulating fetal RNA was also found in maternal blood by Poon *et al.* in 2000 [8]. Since then, other fetal/placental-specific cfRNAs were also reported including human placental lactogen, the β-subunit of human chorionic gonadotropin, and corticotrophin-releasing hormone [9]. These findings have implicated cfRNAs as blood biomarkers for the screening or diagnosis of diseases like cancer and the monitoring of therapy. It also provides the opportunity for early, noninvasive prenatal genetic testing.

When employing cfRNA, however, it is important to minimize release of cellular RNA following blood draw since cfRNA targets present at low quantities [10]. Pre-analytical conditions can affect the release of background RNA into plasma, decreasing the proportion of specific cfRNA targets and masking their detection in downstream applications. Therefore, it is necessary to address pre-analytical issues that arise during the time between blood draw and RNA isolation. These include delays in blood processing, specimen storage temperature, agitation of the samples during transport and shipping. Such conditions may cause cellular RNA release from lysed nucleated blood cells and subsequently alter cfRNA levels circulating in plasma. Thus, in order to obtain reproducible cell-free gene transcript results, it is essential to standardize pre-analytical phase of blood handling using a feasible blood collection device which is capable of stabilizing cfRNA concentration.

We previously reported that we developed a new blood collection device, Cell-Free RNA BCT™ (BCT). The novel chemical cocktail contained in the new device allows for the stabilization of cfRNA in blood samples at room temperature [11]. The goal of the present study was to evaluate the ability of this new blood collection device to stabilize cfRNA and minimize background RNA release when subjected to pre-analytical variables that can occur during sample storage and shipping. The effect of shipping and storage temperature on plasma cfRNA levels in blood collected into BCT or K_3_EDTA tubes have been determined in this study.

## Methods

### Blood sample collection

This study was approved by the institutional review board of the Methodist Hospital, Omaha, NE, USA and informed consent was obtained from all donors prior to blood draw. Blood specimens were collected from apparently healthy adult donors by standard phlebotomy techniques.

For each experiment, blood samples were drawn into two different blood collection tubes. Control samples were drawn into K_3_EDTA tubes (BD Vacutainer®, Becton Dickinson, Franklin Lakes, NJ) and compared to samples drawn into Cell-Free RNA BCT™ (Streck Inc., Omaha, NE). Blood was mixed immediately after the draw by inverting 10 times each.

### Sample processing

Plasma was separated from blood within 2 h post collection (day 0) or after 3 days as noted. To separate plasma, blood samples were centrifuged at 300 × *g* for 20 min at room temperature. The upper plasma layer was carefully removed without disturbing the buffy coat and transferred to a new tube that was then centrifuged at 5000 × *g* for 10 min. The cell-free plasma was then transferred to a new tube for storage at −80°C until cfRNA isolation.

### cfRNA isolation from plasma

The cfRNA was isolated and purified from plasma using the QIAamp® Circulating Nucleic Acid Kit (Qiagen, Santa Clarita, CA). The manufacturer’s recommended protocol was slightly modified by increasing the duration of the Proteinase K treatment at 60°C from 30 min to 1 h. An on-column DNase treatment step was included to remove DNA and cfRNA was eluted in 60 μl of nuclease free water that was passed over the column two times. RNA was stored at −80°C until use.

### Reverse transcription quantitative real-time PCR (RT-qPCR)

All primers were purchased from Integrated DNA Technologies (IDT) (Coralville, IA). All probes for TaqMan® assays were purchased from Applied Biosystems (Foster City, CA), except for β-actin, which was purchased from IDT. Primers and probe for the quantification by RT-qPCR of c-fos mRNA, β-actin mRNA and 18S rRNA were prepared as previously described [11]. To perform RT-qPCR, a TaqMan® RNA-to-C_T_™ 1-Step Kit was purchased from Applied Biosystems (Foster City, CA). Plasmid DNA constructs were prepared by cloning a DNA fragment into Zero Blunt TOPO (Invitrogen, Carlsbad, CA), with each construct containing a single copy of human c-fos or 18S rRNA. The resulting amplicons had lengths of 67 bp and 105 bp, respectively. These plasmid constructs were used to plot the standard curves except for β-actin, which required a synthetic oligonucleotide (Ultramer™ Oligo) that was purchased from IDT. This produced an 86 bp amplicon.

### Effect of shipping on cfRNA concentration in blood samples

For shipping study, blood was drawn from 10 donors. Blood was drawn from each donor into three 10 mL K_3_EDTA tubes and three 10 mL Cell-Free RNA BCT™ (BCT). One K_3_EDTA tube and one BCT from each donor were processed within 2 h of blood draw. Another K_3_EDTA tube and BCT from each donor were shipped at ambient temperature in a box with a temperature tracking device from Omaha, NE to a laboratory in Springfield, MA and back during the course of two days. The remaining K_3_EDTA tube and BCT from each donor were kept at 22°C for three days and processed with the returned shipped blood tubes. Total cfRNA was isolated from plasma and RT-qPCR was used to quantify mRNAs for c-fos, β-actin and 18S rRNA.

### Effect of storage temperature on cfRNA concentration in blood samples

To study the effect of storage temperature on cfRNA concentration, blood samples were collected from 10 donors into four K_3_EDTA tubes and four BCTs each. For each donor, one K_3_EDTA tube and one BCT were immediately processed to separate plasma from blood by centrifugation. These samples are subsequently referred to as K_3_EDTA initial and BCT initial. The remaining samples were stored at 6°C, 22°C or 30°C for 3 days, respectively. Total plasma cfRNA was extracted and mRNAs for c-fos and β-actin were measured by RT-qPCR.

### Statistical analysis

Statistical analysis was carried out using Microsoft Excel for Office 2007. Paired Student’s t-test was used and *p* < 0.05 was considered statistically significant. Fold change calculations were determined as previously described [12].

## Findings

### Effect of shipping on cfRNA concentration in blood samples

**Figure 1:**
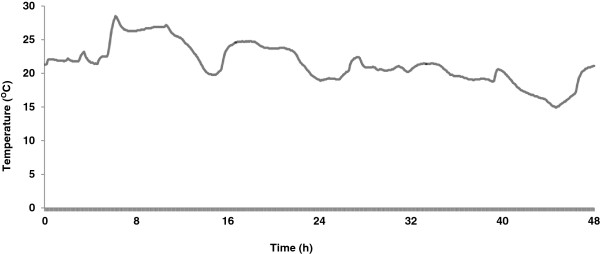
**Shipping temperature record.** A digital temperature logger was included inside the sample box to monitor the temperature during transportation from Omaha NE to Springfield MA and back. The samples were shipped at ambient temperature without any gel packs via FedEx® overnight delivery. As shown in the graph, the samples were retuned back to Omaha NE in approximately 48 h with an exposed temperature range of 14.9 – 28.5°C.

To determine the effect of transportation on cell-free RNA analysis, we collected parallel blood samples from healthy donors into the novel sample collection tubes and control K_3_EDTA tubes. As described previously, blood specimens were either shipped from Omaha NE to a laboratory in Springfield MA and back or not shipped and left at 22°C. Temperature inside the sample box was monitored during the shipment using a digital temperature logger (Omega Nomad, Omega Engineering, Stamford, CT). The samples shipped at ambient temperature were exposed to temperatures ranging from 14.9 to 28.5°C with an average of 21.5°C during the round trip shipping (Figure 1).

**Figure 2:**
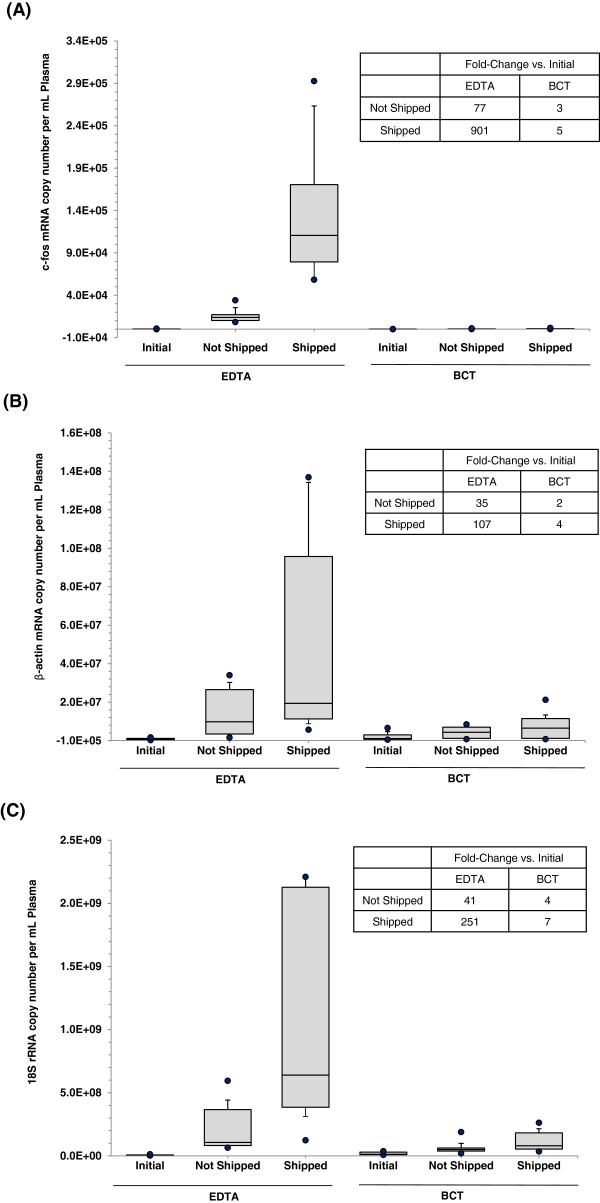
**Effect of shipping on cfRNA concentration in blood samples.** Blood was drawn into K_3_EDTA tubes and BCTs and either shipped round trip to a lab in Springfield MA or not shipped (n = 10). On day 3, plasma from shipped and non-shipped samples was isolated and cfRNA concentrations were determined by measuring c-fos mRNA **(A)**, β-actin mRNA **(B)** and 18S rRNA **(C)** using RT-qPCR. Overall, the BCTs prevent cfRNA concentrations from significant increases as compared to the K_3_EDTA tubes during transportation and room temperature storage. Box plots show the median (line inside the box) and 75th and 25th percentiles (limits of the box). The upper and lower error bars indicate the 90th and 10th percentiles, respectively. The upper most and lower most dots indicate the maximum and minimum values.

Three days post blood draw, both shipped and not shipped blood specimens were processed for cell-free RNA analyses and compared with initial samples which were processed immediately after blood collection (within 2 h). Figure 2A shows the results of shipping of blood when drawn into K_3_EDTA tubes on the cell-free c-fos mRNA concentration. Initially, the median cell-free c-fos mRNA concentration was found to be 92 copies per mL of plasma (copies/mL) that increased markedly in not shipped (stored at room temperature for 3 days) and shipped blood samples. Compared to the initial value, statistically significant increases were observed in the cell-free c-fos mRNA concentration in not shipped (77-fold increase, p = 0.0002) and shipped (901-fold increase, p = 0.0056) blood collected into K_3_EDTA tubes. Figure 2A also illustrates the effect of shipping of blood drawn into BCTs on the cell-free c-fos mRNA concentration in blood. Here, compared to the initial value, there were only slight increases in the cell-free c-fos mRNA concentration in not shipped (3-fold increase, p = 0.17) and shipped (5-fold increase, p = 0.024) blood collected into BCTs. Analysis of β-actin mRNA and 18s rRNA also showed results similar to c-fos mRNA. Figure 2B shows the results of shipping of blood when drawn into K_3_EDTA tubes on the cell-free β-actin mRNA concentration. Compared to initial β-actin mRNA concentration, large increases were observed in the cell-free β-actin mRNA concentration in non-shipped (35-fold increase, p = 0.053) and shipped (107-fold increase, p = 0.023) blood collected into K_3_EDTA tubes. Figure 2B also shows the results of shipping of blood drawn into BCTs on the cell-free β-actin mRNA concentration. In BCTs, compared to initial value, there were only slight increases in the cell-free β-actin mRNA concentration in not shipped (2-fold increase, p = 0.003) and shipped (4-fold increase, p = 0.01) blood. Figure 2C illustrates the results of shipping of blood drawn into K_3_EDTA tubes on the cell-free 18S rRNA concentration. Compared to initial 18S rRNA concentration, statistically significant increases were observed in the cell-free 18S rRNA concentration in not shipped (41-fold increase, p = 0.006) and shipped (251-fold increase, p = 0.009) blood collected into K_3_EDTA tubes. Figure 2C also shows the results of shipping of blood drawn into BCTs on the cell-free 18S rRNA concentration. BCTs showed slight increases in the cell-free 18S rRNA concentration in not shipped (4-fold increase, p = 0.004) and shipped (7-fold increase, p = 0.002) blood.

### Effect of storage temperature on cfRNA concentration in blood samples

**Figure 3:**
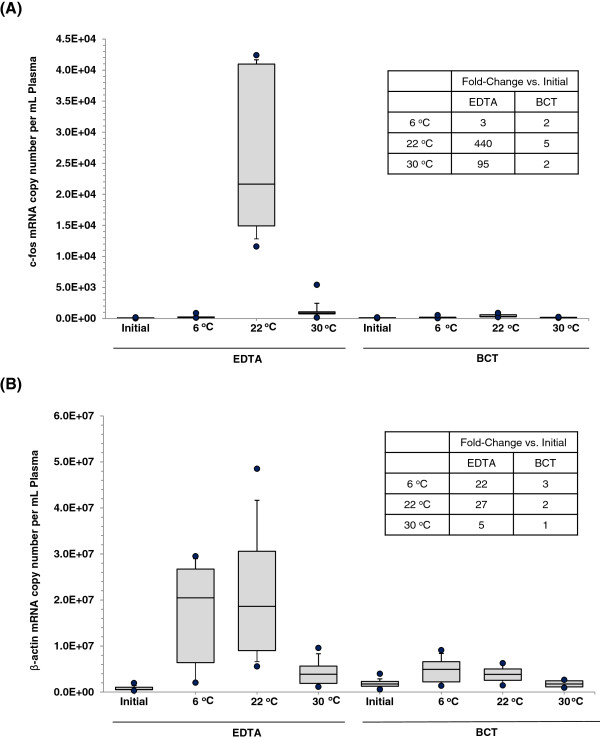
**Effect of storage temperature on cfRNA concentration in blood samples.** Blood was drawn into K_3_EDTA tubes and BCTs (n = 10) and incubated at 6°C, 22°C or 30°C for 3 days, respectively. Plasma was then separated and cfRNA isolated. The cell free mRNAs for c-fos **(A)** and β-actin **(B)** were quantified by RT-qPCR. A significant increase in mRNA copy numbers for c-fos and β-actin was observed in blood stored in K_3_EDTA tubes at 6°C, 22°C and 30°C for 3 days, whereas samples stored in BCTs only showed a slight increase. Box plots show the median (line inside the box) and 75th and 25th percentiles (limits of the box). The upper and lower error bars indicate the 90th and 10th percentiles, respectively. The upper most and lower most dots indicate the maximum and minimum values.

To demonstrate the effect of temperature on cfRNA levels in blood drawn into K_3_EDTA tubes and BCTs, samples were stored at 6°C, 22°C or 30°C for 3 days. After three day incubation period, all blood samples were processed for cell-free RNA analyses and compared with initial samples which were immediately processed after blood collection. As shown in Figure 3A, blood drawn into K_3_EDTA tubes and stored at 6°C, 22°C or 30°C showed 3-fold, 440-fold and 95-fold increase in cell-free c-fos mRNA concentration respectively. However blood drawn into BCTs and stored at 6°C, 22°C or 30°C showed only a slight increase (2-fold, 5-fold and 2-fold respectively) in cell-free c-fos mRNA concentration (Figure 3A). Figure 3B shows the effect of different temperatures on cell-free plasma β-actin mRNA concentration. Blood in K_3_EDTA tubes stored at 6°C, 22°C or 30°C showed 22-fold, 27-fold and 5-fold increase in β-actin mRNA concentration respectively. Blood in BCTs stored at 6°C, 22°C or 30°C showed no significant increase in β-action mRNA concentration.

## Discussion

Noninvasive molecular assays based on cell-free nucleic acid markers circulating in blood are becoming increasingly important to medical diagnostics. Short-term storage or transportation of blood samples from the site of phlebotomy to another facility is commonly required during assay development or clinical application, e.g. multicenter clinical trials and shipping of samples from remote clinics to a central medical laboratory. These pre-analytical variables might compromise the measurement accuracy of molecular targets such as cell-free nucleic acids. In our previous study we had demonstrated that blood drawn into BCTs can be stored at room temperature for up to 3 days without compromising the original quantity and quality of cfRNA [11]. Based on these studies, we hypothesized that BCTs could be used for transportation of blood samples to a centralized facility for blood processing and cfRNA analysis. The present study provides experimental evidence to back this hypothesis. In this study we also explored the best temperature range that the BCTs would perform well by stabilizing cfRNA present in a blood sample. We found that BCT is able to stabilize cfRNA and minimize background RNA release during shipping and storage temperature fluctuations, whereas, as expected, the control K_3_EDTA tubes failed to maintain circulating cell-free RNA concentrations under the same conditions.

Shipping blood specimens prior to cfRNA extraction introduces some pre-analytical variations including agitation and exposure to temperature changes during transportation. The amount of agitation is difficult to control and it may significantly influence accuracy of nucleic acid-based assays. Shaking could disrupt nucleated blood cell integrity and cellular RNA will release into plasma contaminating true cfRNA. In addition, it has been suggested that cfRNA survives in RNase-rich plasma through a protective mechanism, i.e. within lipoprotein vesicles such as apoptotic bodies [13]. Potent enzymes may secret or leak out of cells after shaking and destroy these vesicles resulting in degradation of endogenous circulating RNA. Either increase in background RNA or reduction in original cfRNA will affect test results. In this comparative study, blood was collected into un-stabilized control tubes and BCT tubes containing stabilizing reagents for both cells and cfRNA. To protect samples, tubes were packed in clam shells and then shipped in a Styrofoam box which underwent a 48-h round trip at ambient temperature. Our plasma RNA analysis indicates that cells in BCT withstood shipping conditions much better than those shipped in K_3_EDTA tubes. The BCT showed relatively stable cfRNA concentrations before and after shipping, whereas K_3_EDTA showed dramatic increases in cfRNA under shipping conditions. This suggests nucleated cell disruption occurred in blood samples that were shipped in K_3_EDTA, which led to cellular RNA release, but this was minimized in stabilized samples.

Another common post-phlebotomy variable which needs to be taken into account is variation in sample storage temperature. In current hematology practice, it is not uncommon that blood specimens are delivered to the clinical or research laboratory after a significant post-collection interval. For instance, this interval may exceed 48–72 h on weekends. These specimens are usually kept in a refrigerator or even exposed to room temperature during transportation prior to processing. Cell-free nucleic acid-based assays are presumably more sensitive to storage temperature than these routine hematology analyses, e.g. complete blood count test. Membrane rigidity of blood cells is expected to increase as temperature is lowered [14]. A portion of cells may become fragile and weak. These cells might readily secret cellular RNA into plasma and cause changes in cfRNA concentration during cfRNA processing, e.g. centrifugation. On the other hand, high temperature can also cause problems by overheating and thus deteriorating ex vivo blood cells. In the shipping study mentioned above, the box shipped at ambient temperature kept a relatively stable temperature around 20–24°C during the course of shipment. Although there was temperature elevation up to 29°C, it only occurred for a short period of time. Therefore, we studied the effect of various storage temperatures on the cfRNA concentration of blood drawn into K_3_EDTA and BCT for a longer time. Following blood draw, samples were incubated at 6°C, 22°C or 30°C for 3 days. For all storage temperatures, we observed larger increases in plasma mRNAs in unpreserved K_3_EDTA blood samples as compared to stabilized BCT blood samples. Our blood smear examination under a microscope revealed that leukocytes are well preserved in BCT after incubation. In contrast, a significant number of leukocytes in K_3_EDTA samples was degraded or broken after being incubated at 22°C or 30°C, resulting in release of considerable amount of cellular RNA into plasma. The fold-change is smaller at 30°C than 22°C, which might be explained by the possibility that released RNA and/or cfRNA is degraded more at a higher temperature because the activity of RNase enzymes could be higher at 30°C than 22°C or more such enzymes are released at 30°C. More interestingly, cell-free mRNA concentrations also increase at 6°C in K_3_EDTA tubes, though very few degraded cells have been observed in the smear preparation. This could be due to possible changes in rigidity and permeability of cell membrane. Alternatively, the stress of low temperature might alter gene expressions and thereafter affect secretion of these genes. Taken together, our data demonstrates the ability of the new blood collection device to stabilize cfRNA levels and minimize background RNA release across a broad temperature range for an extended period of time.

Cell-free nucleic acid has the potential to make a significant impact in non-invasive molecular diagnostics. Reports on possible clinical utility of cfRNA are growing [15-17]. Establishing standard protocols is essential for accurate measurements of cfRNA for assay development and clinical practice. Thus, streaming sample collection, transportation and storage is of great significance. To our knowledge, this is the first study focusing on the possibility of shipping and storing unprocessed blood samples for cfRNA analysis. Previous similar investigations have only been done on cellular RNA [18]. The shipping experiment described in this study has not been performed in extremely cold or hot conditions. Therefore, care should be taken to ensure blood specimens are better not exposed to extreme temperatures during shipping. For example, including gel packs in the shipping box may be helpful. When blood samples drawn into BCTs were stored at refrigerated temperature for 3 days, there was a slight hemolysis in the blood sample. A high degree of hemolysis occurred when BCT blood samples were stored at or above 30°C. Therefore, for optimum results, it is suggested that samples collected in BCTs should be stored and/or transported at temperatures between 18–25°C and should be processed within 3 days after phlebotomy, because the novel chemical cocktail has been designed to perform optimally at this temperature range. The effect of potential interfering substances in blood such as hemoglobin, glucose and lipid on BCT performance is worth being investigated, although we have not observed any inconsistencies with random donors. Another limitation of this study is that we only tested several cfRNAs. More cell-free RNA species may need to be examined and validated when using BCTs, especially disease-associated cfRNAs.

In conclusion, our present study demonstrates that cfRNA concentration in whole blood is unstable after transportation or storage when samples are collected in traditional K_3_EDTA tubes. The newly developed blood collection tube with stabilizing agents well maintains the cfRNA level and is suitable for blood shipment and storage prior to processing. Using this new blood collection device, shipping or *ex vivo* storage of blood samples for up to 3 days become possible, allowing flexibility for offsite blood draws to be sent to the facilities capable of downstream analysis of cfRNA without prior centrifugations or cryopreservation.

## Abbreviations

cfRNA: Cell-free RNA; BCT: Cell-Free RNA BCT™; RT-qPCR: Reverse transcription quantitative real-time PCR.

## Competing interests

TW declares that no conflicts of interest exist. JQ & MRF are full time employees of Streck Inc. MRF has a patent dealing with the stabilization of cell-free RNA in blood.

## Authors’ contributions

JQ participated in the experimental design, performed the laboratory work, carried out data and statistical analyses, interpreted the results, drafted the manuscript and prepared the final version of the manuscript. TW was responsible for IRB of blood sample collection, reviewed the manuscript. MRF conceived the study, participated in its design, interpreted the results and revised the manuscript. All authors have read and approved the final manuscript.
